# A Priori and a Posteriori Dietary Patterns in Women of Childbearing Age in the UK

**DOI:** 10.3390/nu12102921

**Published:** 2020-09-24

**Authors:** Karim Khaled, Vanora Hundley, Orouba Almilaji, Mareike Koeppen, Fotini Tsofliou

**Affiliations:** 1 Department of Rehabilitation & Sport Sciences, Faculty of Health & Social Sciences, Bournemouth University, Bournemouth BH1 3LT, UK; Khaledk@bournemouth.ac.uk (K.K.); s5003457@bournemouth.ac.uk (M.K.); 2 Centre for Midwifery, Maternal & Perinatal Health, Faculty of Health & Social Sciences, Bournemouth University, Bournemouth BH1 3LT, UK; vhundley@bournemouth.ac.uk; 3 Department of Medical Science and Public Health, Faculty of Health & Social Sciences, Bournemouth University, Bournemouth BH1 3LT, UK; oalmilaji@bournemouth.ac.uk

**Keywords:** dietary patterns, diet quality, women, childbearing age, a priori, a posteriori, physical activity, socio-demographic, obesity

## Abstract

Poor diet quality is a major cause of maternal obesity. We aimed to investigate a priori and a-posteriori derived dietary patterns in childbearing-aged women in UK. An online survey assessed food intake, physical activity (PA), anthropometry and socio-demographics. An a priori defined diet quality was determined via Mediterranean diet (MD) adherence score and Exploratory Factor Analysis (EFA) derived dietary patterns (DPs). Multiple linear regression explored associations between DPs with anthropometric measures, PA and socio-demographics. Participants (*n* = 123) had low-to-medium MD adherence (average MD-score: 4.0 (2.0)). Age was positively associated with higher MD adherence (X^2^ (2) = 13.14, *p* = 0.01). EFA revealed three DPs: ‘fruits, nuts, vegetables and legumes’ (“Vegetarian-style” DP); ‘sweets, cereals, dairy products and potatoes’ (“Dairy, sweets and starchy foods” DP); and ‘eggs, seafood and meats’ (“Protein-rich” DP). “Vegetarian-style” DP was positively associated with higher maternal educational level (*p* < 0.01) and PA (*p* = 0.01), but negatively with white ethnicity (*p* < 0.01). “Dairy, sweets and starchy foods” DP was positively associated with white ethnicity (*p* = 0.03) and negatively with age (*p* = 0.03). “Protein-rich” DP was positively associated with age (*p* < 0.001) and negatively with PA (*p* = 0.01). A poor diet quality was found among childbearing-aged women; notably in the younger age category, those of white ethnicity, that were more physically inactive and with a lower socioeconomic background.

## 1. Introduction

Obesity rates have been continuously growing in the last decade leading to adversely impact public health worldwide [1]. In 2016, more than 1.9 billion individuals aged 18 years and above were overweight (39% of males and 40% of females) and more than 650 million were obese (11% of males and 15% of females) [2]. Evidence indicates that increased obesity is directly linked with chronic diseases, such as Cardiovascular disease (CVD), Type-two diabetes mellitus (T2DM), cancer, hypertension and increased inflammation [3,4]. Importantly, obesity during pregnancy is a serious condition that affects both the mother and the offspring [5] and can cause gestational diabetes, preeclampsia, still birth, caesarean section and congenital abnormalities of the offspring [6,7]. Recent statistics of maternal mortality in the United Kingdom showed that 209 women died during and one month after pregnancy due to obesity-related complications such as heart diseases, blood clots and strokes [8].

The health status of a pregnant woman is underpinned by nutritional health during the various stages of a woman’s life before pregnancy [9]. Good nutrition is crucial in preconception and pregnancy for it contributes directly to the mother’s, and indirectly to the offspring’s, health [10]. The right nutrition during childbearing age contributes to achieving adequate nutrition stores during pregnancy. In general, women of childbearing age are required to ensure adequate amount of vitamins and minerals such as folic acid, Vitamins A and D, calcium, omega-3 and omega-6 poly-unsaturated fatty acids and iron [11,12,13]. A recent analysis of the UK National Diet and Nutrition Survey (*n* = 3238) reported that women of childbearing age have poor intakes of fruits, vegetables, oily fish and fibre, whereas they highly consume saturated fats, sodium and sugars [14]. Therefore, this poor diet quality will have an adverse impact on their metabolic health across lifecycle.

In the UK, around 30% of women of childbearing age are obese (BMI > 30 Kg/m^2^) [2]. Diet and physical activity are the main lifestyle predictors of obesity and a higher diet quality in women of childbearing age is associated with decreased adiposity [15]. Similarly, Gunderson et al. [16] indicated that poorer diet quality was associated with higher abdominal obesity in women of childbearing age, adding evidence to the inverse association between diet quality and adiposity in this age group.

Recently, there is growing consensus that assessing the quality/pattern of individuals’ whole diet can give a more representative insight of their dietary intake compared to single nutrients [17]; dietary patterns can depict real life habits of food intake and pragmatic food choices [18]. In addition, the measurement of dietary patterns also shows joint and holistic exposure to different food groups and offers stronger health impact on individuals’ health than any single component [19]. Indeed, it is challenging to detect and verify true associations between disease and single nutrients due to the complex interactions between nutrients in the meals consumed [20,21].

The Mediterranean Diet (MD) is an established healthy dietary pattern consumed traditionally by people living in the Mediterranean region. The MD is characterised by high consumption of fruits and vegetable, fish and seafood, olive oil as the main source of lipids and a low consumption of meat and dairy products [22]. Moreover, a high adherence to MD was found to have various benefits to women in preconception such as enhanced fertility [23], decreased hypertension [24], improved mental health and reduced depression [25], reduced risk of gestational diabetes [26] and reduced gestational weight gain [27]. In addition to the protective role of the whole diet, some key nutrients that are found in rich amounts in MD such as antioxidants and unsaturated fatty acids can offer anti-inflammatory and cardio-metabolic benefits in women of childbearing age, especially those with underlying conditions such as obesity and Polycystic Ovary Syndrome (PCOS) [28,29]. Following a Mediterranean-style diet has been found to moderate gestational weight gain and the risk of gestational diabetes in obese pregnant women in a multicentre randomised control trial [30]. However, MD adoption in preconception years might be challenging if perceived barriers and facilitators to diet change are not considered in women of childbearing age. Our previous work highlighted key motivators (e.g., appearance, shared behaviours towards diets with partners) and that emphasis should be given in MD as lifestyle rather than a diet, in order to promote MD adherence to this age group [31].

Dietary patterns are determined through “a priori” or “a posteriori” methods. The “a priori” approach entails the estimation of a diet index/score based on previous food and disease association [32]. One commonly applied diet index in epidemiological studies is the Mediterranean Diet Score (MDS) that is used as an “a priori” approach in studies looking on the correlation between MD and non-communicable diseases [21]. The MDS was developed and updated by Trichopoulou et al. [33] to measure the adherence to MD according to the consumption of nine food groups: cereals, vegetables, fruits and nuts, meat, dairy products, seafood, alcohol, legumes and unsaturated fats. The MDS has been applied in several studies and found to be inversely associated with coronary heart disease and cardiovascular disease [17,33]. On the other hand, the “a posteriori” dietary approach uses data-reduction applications through principal component analysis, factor analysis or cluster analysis to derive dietary patterns based on the dietary intake of participants [34]. These techniques derive dietary patterns by grouping highly correlated food groups together using a data reduction method and subjective criteria are used to determine the number of dietary patterns retained [35,36].

Since the a posteriori approach identifies dietary patterns based on the collected empirical data without a dietary hypothesis, it may not accurately resemble the dietary patterns [37]. On the other hand, the a priori approach is restricted by current comprehension and knowledge of diet-disease relationships [18]. Hence, there is not one ultimate approach to assess the overall diet of individuals. Therefore, the use of both approaches in studies of dietary analysis is considered reasonable as it allows a clear evaluation of dietary patterns and underlying associations through data-driven and hypothesis-driven methods [38]. Yet, only few studies have assessed dietary patterns through both approaches and have targeted different age groups, such as infancy [39], childhood [40] and pregnancy [38].

To the best of our knowledge, there is no existing study that has assessed the dietary quality/patterns in women of childbearing age in the UK. In order to lower obesity and its deleterious health consequences among women of childbearing age, it is necessary to explore their diet, which is the main lifestyle predictor of obesity. Therefore, the aim of the present study was to investigate the diet quality/patterns of women of childbearing age in UK using both a priori and a posteriori methods. The secondary aim of the study was to assess the association between diet quality/patterns and physical activity, socio-demographic and adiposity measures among these women.

## 2. Materials and Methods

### 2.1. Study Population and Sampling Procedure

This cross-sectional study was conducted in the United Kingdom and enrolled a convenience sample of women of childbearing age who were students and staff from the nursing department at a UK Higher Education Institution. A multi-section online survey was developed and sent via emails to participants to assess their dietary patterns and other variables (physical activity (PA) levels, socio-demographic characteristics and adiposity measures). Participant information about the scope of the study and eligibility criteria was provided in the recruitment email and on the landing page of the survey. Women were not eligible to participate if they were pregnant or breast feeding, aged less than 18 or above 49 years, suffering from a chronic disease, having food intolerance or allergy, taking medications that affect appetite or seeking to lose or gain weight intentionally. Participation was voluntary, and those who did not meet the inclusion criteria and did not consent were unable to carry on with the online survey; they were directed away from the survey to a page thanking them for their time. Confidentiality was protected by ensuring that the survey did not contain personal identification or information that would identify participants, such as names, emails or IP addresses. A prize draw of £100 Amazon voucher was done as an incentive for completing the survey.

The sample was collected at two time points. In all, 123 healthy women aged 18–49 years took part in the study. All participants gave their informed consent for inclusion before they participated in the study. The study was conducted in accordance with the Declaration of Helsinki, and the protocol was approved by the Ethics Committee of Bournemouth University, Fern Barrow, UK(Ref ID 17949; 14565).

### 2.2. Survey

The survey was created using BOS (Bristol Online Surveys) and consisted of six pages. The first and second pages contained the Participant Information Sheet and the consent form and the last page contained a “thank you” note. The third page of the survey contained socioeconomic questions in addition to adiposity measures. A 100-food item validated semi-quantitative Food Frequency Questionnaire (FFQ) to assess diet patterns was on the fourth page. Page five contained the International Physical Activity Questionnaire (IPAQ) that measured the physical activity levels of participants.

### 2.3. Anthropometric Measurement

Body weight, height and waist circumference were self-reported by women. The BMI was calculated as weight (kg) divided by the square of height (m^2^). Women with BMI greater than 30 Kg/m^2^ were classified as obese, those with BMI between 25 and 30 Kg/m^2^ were classified as overweight, women with a BMI of 18 to 25 Kg/m^2^ were classified as normal weight and those with a BMI of less than 18 Kg/m^2^ were classified as underweight [41]. Waist-to-height ratio (W/Ht) was calculated by dividing waist circumference (cm) by height (cm).

### 2.4. Food Frequency Questionnaire

The FFQ is a validated tool developed by Robinson et al. [42] and it includes 100 food items. Participants were asked how often, on average, they consumed these foods over the previous 3 months. The frequencies were categorised as never, once every 2–3 months, once a month, once a fortnight, 1–2 times per week, 3–6 times per week, once a day or more than once a day. The grams of each food item consumed per day were calculated by multiplying the portion size of each food item by its frequency of consumption divided by seven [33,43].

### 2.5. Physical Activity

The International Physical Activity Questionnaire (IPAQ; short, 7-d recall) was used for assessing the physical activity (PA) of the study subjects. This questionnaire measures three types of activities: walking, moderate intensity and vigorous intensity. PA was expressed as the metabolic equivalent of task (MET)-min/week. According to the calculated MET-min/week, PA levels were categorised into: low, moderate and high [44].

### 2.6. Dietary Patterns

Dietary patterns were explored through the commonly used a priori approach (adherence to Mediterranean Diet) and a posteriori approach (Factor analysis). To measure the diet patterns of participants, the 100 food items of the FFQ were divided based on similarity of their nutrient profiles into 13 food groups as follows: Cereals, legumes, vegetables, fruits and nuts, meat, fish and seafood, dairy products, fat ratio (MUFA+PUFA/SFA), eggs, potatoes, sweets, drinks and miscellaneous (Appendix A). Factor analysis was performed on the 13 food groups in order to explore the dietary pattern of women. The adherence to Mediterranean diet (MD) was derived through a Mediterranean Diet Score (MDS) previously used in a large cohort of adults (European Prospective Investigation into Cancer and Nutrition) [33]. Women who consumed above the present study’s median of nuts and fruits, vegetables (excluding potato), legumes, seafood, cereal and ratio of monounsaturated lipids + polyunsaturated lipids to saturated lipids were given a score of one, whereas women who consumed lower than the median of these food groups were given a score of zero [33,45]. Conversely, when women consumed above the median of processed meat and dairy products, they were given a score of zero, whereas when they consumed lower than the median of these foods, they were given a score of one [33]. The MDS ranges from zero as the lowest adherence to MD to eight as the highest adherence. The score was divided into three categories: less than 3, indicating low adherence to MD, between 4 and 5, indicating medium adherence, and from 6 to 8, indicating high adherence.

### 2.7. Data Analyses

Data were analysed using IBM SPSS statistics version 25 (Chicago, IL, USA). A Shapiro-Wilk test was used to test the normality of the data. Descriptive data are presented as mean values and standard deviations for normally distributed data and median and interquartile range (IQR) for non-normally distributed data. Continuous data were compared between the MD adherence categories (low, medium, high) through a Kruskal-Wallis test. Categorical data were compared between the MD adherence categories (low, medium, high) using a Chi square test. After testing for normality, the data of BMI, WC/HT and Age were not normally distributed, as assessed by Shapiro-Wilk test (*p* = 0.002 for BMI, *p* < 0.001 for W/Ht) and *p* < 0.001 for age in Low MD adherence score; *p* < 0.001 for BMI, *p* = 0.002 for WC/HT and *p* < 0.001 for age in Medium MD Adherence score; *p* = 0.002 for BMI, *p* = 0.065 for WC/HT and *p* = 0.005 for age in High MD adherence score). Consequently, a Kruskal-Wallis H test was conducted to determine if there were differences in BMI, WC/HT and age data between the three MD adherence categories: “Low MD score” (*n* = 48), “Medium MD score” (*n* = 49) and “High MD score” (*n* = 26). Distributions of BMI, WC/HT and age were not similar for all groups, as assessed by visual inspection of a boxplot.

From the food frequency questionnaire, 11 food groups (g/d) were built. The distributions of the various food groups were examined, and proper transformations were carried out to correct any high skewness (>1) in the data. Kaiser-Meyer Olkin (KMO) and Bartlett’s Test measure of sampling adequacy was used to examine the appropriateness of Factor Analysis. The test results showed that the approximate of Chi-square is 184 with 55 degrees of freedom, which was very significant (*p* < 0.00001). The KMO statistic was also large (>0.5). Hence Factor Analysis was considered an appropriate technique for further analysis of the data. To determine the number of factors/diet patterns, a “scree” plot was produced from which the selection of factors was based on: (1) Kaiser-Guttman normalisation rule (choosing factors with eigenvalue greater than (1) and (2) Bend elbow rule. Assuming that the factors were uncorrelated, an orthogonal rotation (varimax) has been used to calculate factors. The minimum significance loading for each factor was considered at a cut off of “>0.3”. For each dietary pattern (factor), simple linear regression models were run to assess the association between the dietary pattern (outcome) and the following variables separately: BMI, WC/HT, PA and socioeconomic factors. When a significant association was found, these predictors were added to a multiple linear regression model along with all other significant predictors of the same diet pattern.

Due to the small size of some categories of socioeconomic factors, the numbers of categories were reduced by merging the close categories together before inclusion in the regression models (marital status (single/divorced, living together/married), education (No qualification/Certificate of Secondary School (CSE)/General Certificate of Secondary School (GCSE), A-level/higher education), ethnicity (white, other), smoking status (smoker, non-smoker), income (below the average, above the average)).

## 3. Results

Table 1 shows the socio-demographic characteristics along with physical activity levels and adiposity measures in the total sample of 123 women by categories of MD adherence (low, medium, high). The assessment of MD adherence showed that low to medium diet quality was highly prevalent among study participants; 39% of women had low MD adherence compared to 21% who had high MD adherence. Approximately 40% of women had medium adherence to MD (Table 1). Participants were mostly single and white and around half of them were below 25 years old and had a BMI > 25 Kg/m^2^ (overweight/obese). Fifty-eight percent of the participants had low-to-moderate PA levels, and 42% had high PA levels. Age was significantly different across the three categories of MD adherence (X^2^(4) = 13.14, *p* = 0.011). Participants aged 18–25 years were more likely to have a low MD adherence (23.7%) than those aged 25–34 years (7.6%) and 35–49 years (7.6%). Median age (25.5 years (IQR 10.0)) increased from Low (mean rank= 50.41), to Medium (mean rank = 59.24), to High (mean rank = 78.22) MD adherence groups. Pairwise Comparisons showed that age was significantly different between Low MD Adherence and High MD Adherence (*p* = 0.004), but not between Low and Medium (*p* = 0.62) or Medium and High (*p* = 0.08) MD Adherence. Moreover, marital status was found to be significantly different across the MD adherence categories (X^2^(6) = 14.25, *p* = 0.027). A higher percentage of single or divorced participants had low and medium MD adherence (25.4% and 26.2%, respectively) compared to high adherence (8.2%). MD adherence was not associated with BMI (*p* = 0.53), WC (*p* = 0.911), WC/HT (*p* = 0.797) and Physical Activity (METs) (*p* = 0.477) (Table 1).

### 3.1. Intake of MD Individual Food Components and MD Adherence

Table 2 lists the MD food components across the different MD adherence groups and shows the MD score of the whole sample. The total MDS was 4.0 and significantly different across MD adherence categories (*p* < 0.001). The cereals and meat intakes did not significantly differ between low and high MD adherence groups. The intakes of legumes, vegetables, fruits and nuts and fish and seafood and the ratio of unsaturated fats to saturated fats (MUFA+PUFA: SFA) along with the MD Score were significantly higher in high MD adherence group than low MD adherence group (*p* < 0.01). The difference in the mean dairy products intake between low MD adherence and High MD adherence was 251 g where the intake was significantly higher in the low MD adherence group than high MD adherence group (*p* < 0.01).

### 3.2. Factor Analysis

**Figure 1:**
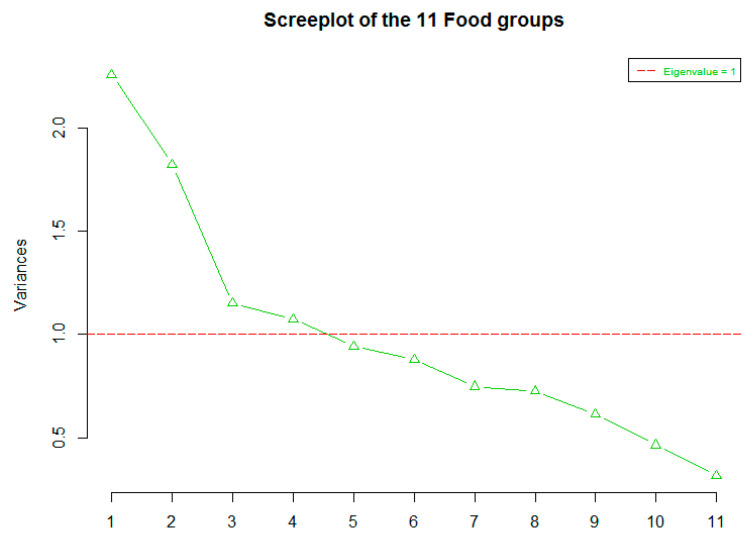
A Scree Plot of the 11 food groups showing the eigenvalues on the y-axis and the number of factors on the x-axis.

The scree plot (Figure 1) shows that the maximum number of factors (dietary patterns) that could be used for the factor analysis is 4 in which eigenvalue ≥1. Using the point where the slope of the curve is clearly levelling off (the “elbow), it is indicated that the number of factors that should be generated by the analysis is 3. Based on the Kaiser Guttman normalisation and bend elbow rules mentioned above, factor analysis revealed three dietary patterns among study participants. The first was labelled “Vegetarian-style dietary pattern” (DP-1) since the factor loadings were high for fruits and nuts, vegetables and legumes. In the second dietary pattern (DP-2), the highest factor loadings were for dairy products, sweets, cereals and potatoes; therefore, DP-2 was labelled “Dairy, sweets and starchy foods dietary pattern” (DP-2). The third dietary pattern (DP-3) was labelled “Protein-rich dietary pattern” since the highest factor loadings were for the eggs, fish and seafood and meat.

The number of factors/dietary patterns retained was three, explaining 48% of the total variance in the data. Assuming that the factors were uncorrelated, an orthogonal rotation (varimax) has been used to calculate factors. Table 3 shows the food groups with the highest factor loadings for the three factors/dietary patterns and the structure of food groups loading in each one of them, respectively. Using a minimum of significance loading on each factor at a cut-off of >0.3, three food groups were found in Factors 1 and 3 (DP-1 and DP-3) and four food groups in Factor 2 (DP-2) (Table 3).

The first multiple regression model (Model 1) was built based on the following formula: DP-1 = $\beta_{0}$ + $\beta_{1}$METs + $\beta_{2}$Income + $\beta_{3}$Ethnicity + $\beta_{4}$mother education. The model showed that the “Vegetarian-style” dietary pattern (DP-1) was positively associated with higher educational level of the mother (A-level/higher education) (*p* < 0.01) (Table 4). Additionally, the model showed a high and significant negative association with white ethnicity (*p* < 0.01) and a significant positive association with the physical activity level of participants (*p* = 0.01). Although the simple regression model has shown that DP-1 was significantly associated with the parental income of participants, only a marginal positive association was found (*p* = 0.09) after adding this predictor to the multiple linear regression. This DP was common among women who were more physically active, from non-white ethnicity and daughters of more/highly educated mothers.

Model 2 was built according to the following formula: DP-2 = $\beta_{0}$ + $\beta_{1}$METs + $\beta_{2}Age$+ $\beta_{3}$Ethnicity. As shown in Table 4, the model found that “Dairy, sweets and starchy foods” dietary pattern (DP-2) had a significant positive association with white ethnicity (*p* = 0.03) but a significant negative association with age (*p* = 0.03). Although the simple regression model showed a significantly negative association between DP-2 and physical activity (METs), a marginal negative association (*p* = 0.06) was found after adding this predictor to the multiple linear regression of DP-2. This DP was common among the white and younger women.

Model 3 was built based on the following formula: DP-3 = $\beta_{0}$ + $\beta_{1}$METs + $\beta_{2}$Age. The model showed that DP-3 (“Protein-rich” dietary pattern) had a positive high association with age (*p* = 0.0008) and a negative significant association with physical activity (*p* = 0.01) (Table 4). This pattern was common among the older and less active women.

## 4. Discussion

Diet quality analysis has been recently trending in nutrition epidemiology research, since it permits the measurement of diet as a whole and describes the food intake of free-living individuals. The objective of this study was to investigate the diet quality of women of childbearing age through both a priori and a posteriori approaches and their associations with adiposity measures, physical activity and socio-demographic characteristics.

A priori evaluation of dietary quality/pattern via the Mediterranean Diet score [33] found an overall moderate adherence to the Mediterranean Diet (MDS = 4.0) in women of childbearing age in UK. This is in line with a recent study among employees in the UK (*n* = 587), which found that most female participants (59%) had a moderate adherence to Mediterranean diet [46]. Boghossian et al. [47] reported similar results; the study assessed the adherence to Mediterranean diet among 248 women of childbearing age in USA and found that 43% of participants were moderate adherers, whereas 35% were low adherers and 22% were high adherers.

The current study found that age was significantly associated with higher diet quality (higher adherence to MD). This has been previously reported in many studies. For example, VanKim et al. [48] found that the age of 100,000 women of reproductive age, who were nurses in the USA, was significantly associated with higher diet quality (assessed through Alternative Healthy Eating Index-2010 (AHEI-2010) and Dietary Approaches to Stop Hypertension index (DASH)). A cross-sectional nutrition survey conducted in Germany by Thiele et al. [49] also showed that participants’ age (2267 women) was associated with higher diet quality (assessed by two indices constructed in the study by authors). Among 4038 Brazilian individuals (adolescents, adults and older adults) from both sexes, it was found that the lowest diet quality, as assessed by the Brazilian Healthy Eating Index, was among the adolescents age group [50]. These results may be explained by the findings of some studies where older participants tended to consume more vegetables, fruits and cereals (contributing to higher diet quality) than younger ones, whereas the latter had higher intake of sweets and unhealthy snacks (contributing to lower diet quality) [51,52]. An in-depth insight of diet quality variation can be obtained if population cohorts can be studies over time and across lifespan; investigating how the diet quality of the present study’s participants will change with time would interestingly show some new results.

The present study found no significant association between adiposity measures and the adherence to MD among participants. This is consistent with the results of two longitudinal studies. The first study followed up a sample of Spanish graduates for two years and four months and found no association between adherence to MD and weight change and BMI [53]. The second study was conducted among 732 Chinese adults aged >35 years (including 385 women) with a follow up duration of 5–9 years and reported no significant correlation between MD adherence and BMI [54]. However, 30% of participants opted out of the study during follow up. These results were also found among 6619 Italian adults (including 3529 women) and 23,597 Greek adults (including 13,985 women), where no significant associations were found between MD adherence and BMI or waist-to-hip ratio after adjusting for confounding factors [55,56]. On the contrary, Boghossian et al. [47] found an inverse association between the adherence to Mediterranean diet and adiposity measures (percent body fat, percent trunk fat and percent leg fat, sum of skinfolds, waist circumference, hip circumference and BMI) among 248 women of childbearing age. However, all the anthropometric measures in that study were measured twice by trained professionals through Dual X-ray Absorptiometry (DXA), which clearly justifies the discrepancy of findings with the present study regarding the association between MD adherence and adiposity. Additionally, three prospective cohort studies of middle-aged men and women reported lower weight gain and obesity among high MD adherers compared to low MD adherers [57,58,59]. A statistically significant negative association was also reported among Spanish adults between BMI and adherence to MD; however, the author did not account for monounsaturated fats (which is an important element in deriving the MDS) [60]. Romaguera et al. [61] assessed the adherence to MD and the association with BMI and WC among 500,000 adults (70% women) from 10 European countries and found inconsistent results with the present study. The study found an inverse association between MD adherence and adiposity measures; however, the author reported some limitations, such as selection and recall biases in addition to measurement errors in food intake assessment. The diversity in results between these studies and the present study can be interpreted by differences in study designs, the way of measuring the anthropometric variables, the target sample included and the way diet was assessed in each study. All studies measured the adherence to MD through the MDS [33]; however, most of them did not particularly target women of childbearing age, but rather the adult population (20–70 years old) [57,58,59]. Additionally, the sample size of the present study is much smaller than the aforementioned studies. For instance, 123 women participated in the present study versus 248 women in Boghossian et al. [47], *n* = 17,238 women in Mendez et al. [58], and *n* = 351,730 women in Romaguera et al. [61]. Although Boghossian et al. [47] assessed the adiposity-diet association among women of childbearing age, the author used 24-h recalls to assess dietary intake and DXA to accurately assess adiposity measures, which had a huge impact on the study results (versus FFQ and self-reported adiposity measures in the present study). Similarly, in Schroder et al. [60], trained personnel measured the weight and height of participants through calibrated scales before deriving their BMI. While the present study had a cross-sectional design, Beunza et al. [57], Mendez et al. [58] and Romaguera et al. [59] had a prospective longitudinal design, which allowed the authors to report their results based on the participants’ weight change over time. Studies assessing the association between adiposity and diet quality should use standardised tools (such as DXA) to accurately measure adiposity and recruit a large sample size to detect latent associations.

The adherence to MD was not associated with participants’ physical activity level in the present study. Evidence in the literature about this association is scarce and reports opposite results. A study among 1175 women of childbearing age in Spain showed a significantly positive association between MD adherence (MDS) and sedentary lifestyle (assessed by Paffenberger physical activity questionnaire) [62]. Similar findings were reported among 2260 women from the SUN prospective cohort, where participants who were more active had a higher MD adherence (*p* < 0.001) [63]. These studies have quite bigger sample sizes than that of the present study, and that might be an explanation for discrepancies in findings. A larger sample size and more credible methods for measuring physical activity (such as Accelerometers) are needed in epidemiological studies to produce robust results [64].

The present study derived three dietary patterns through a-posteriori approach: Vegetarian-style DP (fruits, nuts, vegetables and legumes), Dairy, sweets and starchy foods DP (sweets, cereals, dairy products and potatoes) and Protein-rich DP (eggs, seafood and meats). Similar results were reported among 3347 pregnant women in the Netherlands, where three major diet patterns were derived containing “vegetable, oil and fish”, “nuts, high fibre cereals and soy”, and “margarine, sugar and snacks” patterns [38]. A recent study done among UK adults (*n* = 2083, 56.7% females) identified four dietary patterns through Principal Component Analysis (PCA): “snack, fast food and fizzy drinks”, “fruits, vegetables and oily fish”, “meat, potatoes and beer”, and “sugary foods and dairy” [65]. Crozier et al. [66] assessed a posteriori dietary patterns among 6125 women of childbearing age (aged 20–34 years) in the UK. Two main patterns were derived: a prudent diet (high in fruits, vegetables, whole meal bread, yoghurt, pasta, rice and cereals) and a high-energy diet (meat, fish, eggs, cakes, biscuits, fat and potatoes). Therefore, the dietary patterns of UK women of childbearing age are basically divided into several patterns that might comprise healthy (high intake of fruits, vegetables, fibres and fish) and/or unhealthy (sugar, snacks and high fat foods) food intakes. The present study found that physical activity (METs) was negatively associated with “Dairy, sweets and starchy foods” and “Protein-rich” dietary patterns but positively with the “Vegetarian-style” dietary pattern. The Vegetarian-style dietary pattern was positively associated with socioeconomic status, but negatively with white ethnicity. Age was negatively associated with “Dairy, sweets and starchy foods” dietary pattern and positively with “Protein-rich” dietary pattern. In the same context, Robert et al. [65] found that the “snack, fast food and fizzy drinks” dietary pattern was linked negatively with age but positively with BMI, urinary sodium and smoking. They also reported that the “fruit, vegetables and oily fish” dietary pattern was linked positively with non-white race, age and income, but negatively with smoking, BMI and urinary sodium. These findings propose that women of childbearing age in the UK have various dietary patterns, each with different diet quality, and are associated with socio-demographic and lifestyle factors. This is worth noting since lifestyle factors (such as physical activity) along with socio-demographics (such as age, ethnicity and income) are directly associated with health-related outcomes, independent of food intake [67]. Moreover, the findings suggest that using an a posteriori approach is crucial to investigate and describe the dietary patterns of UK women of childbearing age and provides useful information to public health nutrition policies and research. The use of a priori derived dietary patterns also highlights the significance of using these methods of analysis in exploring the diet quality/pattern as a complex and multi-dimensional variable.

### Strengths and Limitations

This study is the first to assess the diet quality/patterns of women of childbearing age in the UK using both a priori (adherence to Mediterranean Diet) and a posteriori (Factor analysis) dietary analysis approaches. This is of high importance, as it allows comprehensive and rigorous evaluation of dietary quality/patterns and their associations through both data-driven and hypothesis-driven methodologies [38]. We excluded women who had chronic diseases or food intolerance and those taking appetite suppressants or following weight-losing dietary programs as these might bias the results since their pattern of food intake would be abnormally affected in some way by these conditions. Moreover, the study reported data of various confounding factors (such as physical activity, socio-demographics and adiposity measures) and adjusted for these variables in the data analysis. Additionally, the tools used to measure all the different variables were validated and standardised [42,68,69,70].

On the other hand, we acknowledge some limitations in the present study. The participants of the study were from the nursing department only, and hence, results cannot be generalised to the whole childbearing aged female population. The cross-sectional design of the study made it difficult to draw a definitive conclusion about the diet quality of women of childbearing age in the UK and its association with the measured variables. Additionally, the current sample size (*n* = 123) can be deemed as low for a cross-sectional study and future studies should be more representative of the whole of the child-bearing-aged women and include hard-to-reach-groups. Furthermore, almost all variables were self-reported, which could have led to inaccurate reporting by participants. Although the food frequency questionnaire used in the study was validated and widely used, it did not assess alcohol intake, which might have played a role in determining the diet quality in the present study. Interestingly, the Mediterranean Diet Score has its own limitations, just like all other diet quality indices. For example, there is variability in the medians used to identify cut-off points for scoring of food components and in food distribution among different populations [71]. This is occurring because each study will have a different median (cut-off point for MD scoring) based on the data of food-groups reported by the study participants [71,72].

In conclusion, our findings indicate that actions should be undertaken to encourage healthier food choices and physical activity, and that nutrition education should be a major consideration for health care programs targeting women at a national level. In the present study’s specific setting (the UK), awareness about following a healthy lifestyle with emphasis on diet, healthy weight and pragmatic physical activities should be promoted among women of childbearing age within the existing public health guidelines and policies. Knowing the factors associated with low diet quality can be important in order to implement health-improving programs that enhance healthy dietary habits among the overall population, and specifically women of childbearing age. Promoting nutritional education and healthy dietary habits is a societal and political/governmental requirement; thus, there is a need for interventions with combined efforts [73]. Women of childbearing age are an important age-group to target with such preventative measures, as this will enhance their overall health as individuals, future mothers and for their offspring. Therefore, the childbearing age can be deemed ideal for interventions to change lifestyle behaviours towards healthier eating and PA habits, considering that women in this age are highly motivated and willing to accept behavioural changes [74]. To implement effective dietary interventions towards healthier dietary patterns such as a Mediterranean-style diet, we should consider that younger age, white ethnicity, lower socioeconomic characteristics (pertinent to lower maternal education) and physical inactivity might be associated with lower diet quality in women of childbearing age.

## Figures and Tables

**Table 1:** Socio-demographic and physical characteristics of participants (*n* = 123).

Participants’ Characteristics	Total Sample	Mediterranean Diet Adherence Categories			*p* Value
		Low MDS (0–3)	Medium MDS (4–5)	High MDS (6–8)	
	N (%)	48 (39)	49 (40)	26 (21)	
**Physical and lifestyle Characteristics**					
**Age (years)** #	25.5 (10.0)	23.0 (10.0)	24.5 (8.3)	28.0 (10.5)	0.006
Age (years) *					
18–24	56 (47.4)	28 (23.7)	24 (20.3)	4 (3.4)	
25–34	38 (32.2)	9 (7.6)	16 (13.6)	13 (11.0)	0.01
35–49	24 (20.3)	9 (7.6)	9 (7.6)	6 (5.1)	
**BMI (kg/m^2^)** #	24.3 (6.8)	24.2 (6.8)	24.3 (6.3)	25.0 (7.4)	0.53
BMI *					
Overweight/obese	59 (48.0)	25 (20.4)	21 (17.1)	13 (10.6)	0.485
Normal Weight	64 (52.0)	23 (18.7)	28 (22.8)	13 (10.6)	
**Waist Circumference (cm)** #	71.6 (48.0)	71.1 (43.0)	71.6 (51.0)	80.0 (65.0)	0.911
Waist Circumference *					
Overweight/obese	64 (62.7)	26 (25.5)	25 (24.5)	13 (12.7)	0.658
Normal weight	38 (37.3)	12 (11.8)	17 (16.7)	9 (8.8)	
**Waist to Height Ratio** #	0.45 (0.30)	0.44 (0.28)	0.45 (0.30)	0.48 (0.41)	0.797
Waist to Height Ratio *					
Overweight/obese	31 (30.4)	12 (11.8)	10 (9.8)	9 (8.8)	
Normal weight	71 (69.6)	26 (25.5)	32 (31.4)	13 (12.7)	0.361
**Physical Activity (MET)** #	2260 (3499.5)	1443 (2711.25)	2515 (4690.5)	2055 (3049.5)	0.477
Physical Activity *					
Low (<600 MET minutes/week)	24 (19.5)	15 (12.2)	6 (4.9)	3 (2.4)	
Moderate (>600 MET minutes/week)	47 (38.2)	15 (12.2)	20 (16.3)	12 (9.8)	0.126
High (>3000 MET minutes/week)	52 (42.2)	18 (14.6)	23 (18.7)	11 (8.9)	
**Socio-demographic characteristics**					
Father’s Educational level *					
No qualifications	14 (11.5)	5 (4.1)	6 (4.9)	3 (2.5)	
Certificate of secondary education	13 (10.6)	6 (4.9)	6 (4.9)	1 (0.8)	
O-level or GCSE	39 (32)	19 (15.6)	10 (8.2)	10 (8.2)	0.471
A-level school examination	15 (12.3)	5 (4.1)	8 (6.6)	2 (1.6)	
Higher education	41 (33.6)	12 (9.8)	19 (15.6)	10 (8.2)	
Mother’s Educational level *					
No qualifications	7 (5.7)	2 (1.6)	4 (3.3)	1 (0.8)	
Certificate of secondary education	7 (5.7)	4 (3.3)	2 (1.6)	1 (0.8)	
O-level or GCSE	46 (37.4)	23 (18.7)	17 (13.8)	6 (4.9)	0.377
A-level school examination	11 (9)	4 (3.3)	5 (4.1)	2 (1.6)	
Higher education	52 (42.3)	15 (12.2)	21 (17.1)	16 (13)	
Household income (Pound/year) *					
<£13,000	17(13.8)	9 (7.3)	6 (4.9)	2 (1.6)	
£13,000 to £23,400	28 (22.8)	13 (10.6)	11 (8.9)	4 (3.3)	0.528
£23,400 to £33,800	38 (30.9)	16 (13)	14 (11.4)	8 (6.5)	
£33,800 to £52,000	29 (23.6)	8 (6.5)	13 (10.6)	8 (6.5)	
>£52,000	11 (8.9)	2 (1.6)	5 (4.1)	4 (3.3)	
Marital Status *					
Single or divorced	73 (59.8)	31 (25.4)	32 (26.2)	10 (8.2)	0.027
Married or living together	49 (40.2)	16 (13.1)	17 (14.0)	16 (13.1)	
Smoking *					
Smoker	26 (21.5)	11 (9.1)	9 (7.4)	6 (5)	0.616
Non-smoker	95 (78.5)	37 (30.6)	39 (32.2)	19 (15.7)	
Occupation *					
Student	117 (95.1)	45 (36.6)	48 (39.0)	24 (19.5)	0.391
Associate professional or professional	2 (1.6)	0 (0)	1 (0.8)	1 (0.8)	
Clerical, sales or service worker	4 (3.2)	3 (2.4)	0 (0)	1 (0.8)	
Ethnicity *					
White	71 (58.1)	32 (26.2)	27 (22.1)	12 (9.8)	0.353
Other	51 (41.7)	16 (13.1)	21 (17.2)	14 (11.4)	

METs-h/wk: Metabolic Equivalents of Tasks-hours per week, BMI: Body Mass Index.,GCSE: General Certificate of Secondary Education, O-level: Ordinary level. *p* values were derived through a Chi-square test of Independence to display differences in socio-demographic, physical activity and adiposity statuses of participants across the three Mediterranean diet (MD) adherence categories. The differences between Median (IQR) of physical and lifestyle characteristics were explored with Kruskal-Wallis test and posthoc pairwise comparisons. * represents *N* (%). # represents Median (IQR). MDS: Mediterranean Diet Score.

**Table 2:** Intake of MD individual food components across MD adherence categories.

MD Food Components (Median (IQR))	Total Sample (*n* = 123)	MD Adherence Categories			*p* Value
		Low MDS (0–3)	Medium MDS (4–5)	High MDS (6–8)	
Cereals (g/d)	280.4 (239.4)	270.0 (237.3)	279.1 (258.7)	316.1 (199.4)	0.748
Legumes (g/d)	53.6 (87.9)	30.7 (45.3)	102.9 (68.6)	97.5 (107.1)	<0.001 *
Vegetables (g/d)	603.5 (432.4)	381.5 (288.8)	708.9 (395.9)	809.3 (376.0)	<0.001 *
Fruits & Nuts (g/d)	350.2 (364.1)	217.9 (255.0)	350.9 (322.3)	568.8 (453.9)	<0.001 *
Meat (g/d)	125.3 (139.9)	137.2 (165.1)	122.3 (109.5)	112.1 (156.6)	0.696
Fish & seafood (g/d)	32.1 (71.9)	16.0 (25.3)	42.2 (69.5)	67.0 (79.5)	<0.001 *
Dairy products (g/d)	343.6 (334.1)	504.0 (440.3)	311.9 (295.9)	253.9 (243.5)	<0.001 *
(MUFA+PUFA)/SFA	1.58 (0.63)	1.42 (0.29)	1.60 (0.70)	2.07 (0.76)	<0.001 *
MD Score	4.0 (2.0)	2.5 (1.0)	5.0 (1.0)	6.0 (1.0)	<0.001 *

*p* values were derived through Kruskal-Wallis Test. * indicates significant differences across the three MD adherence categories. MD: Mediterranean Diet, IQR: Interquartile Range, MUFA: Mono-unsaturated fatty acids, PUFA: Poly-unsaturated fatty acids, SFA: Saturated fatty acids, MDS: Mediterranean Diet Score.

**Table 3:** Varimax rotated factor loadings for the three factors of the 10 food groups derived from the food frequency questionnaire.

Food Groups	Factor 1	Factor 2	Factor 3
Cereals (g/d)		0.83	
Legumes (g/d)	0.48		
Vegetables (g/d)	0.98		
Fruits and Nuts (g/d)	0.40		
Meat (g/d)			0.33
Fish and seafood (g/d)			0.79
Eggs (g/d)			0.42
Potatoes (g/d)		0.44	
Dairy Products (g/d)		0.36	
Sweets (g/d)		0.39	

The association between a posteriori-derived diet patterns and adiposity measures, physical activity and socioeconomic factors.

**Table 4:** Regression models showing the association between a posteriori-derived diet patterns and adiposity measures, physical activity and socioeconomic factors.

Model	Predictor	Coefficient Estimate	*p* Value
1 (Vegetarian-style dietary pattern)	Intercept	−0.3	0.2
	Physical Activity (METs- h/wk)	0.00003	0.01
	Parental Income (above average)	0.3	0.09
	Ethnicity (white)	−0.5	<0.01
	Mother’s education (A-level/higher)	0.5	<0.01
2 (Dairy, sweets and starchy foods dietary pattern)	Intercept	0.5	0.1
	Physical Activity (METs- h/wk)	−0.00002	0.06
	Ethnicity (white)	0.4	0.03
	Age	−0.02	0.03
3 (Protein-rich dietary pattern)	Intercept	−0.8	<0.01
	Physical Activity (METs- h/wk)	−0.00002	0.01
	Age	0.03	<0.001

METs-h/wk: Metabolic Equivalents of Tasks-hours per week.

## Appendix A

**Table A1:** Classification of food items according to nutrient profiles.

Food Groups	Food Items
Cereals	White bread, Brown and wholemeal bread/rolls, Crackers and cheese biscuits, Wholemeal and rye crackers, ‘Bran’ breakfast cereals, Other breakfast cereals, Added bran to foods, Brown and white rice, Pasta and dumplings, Pizza quiches/cheese flans, other puddings, Pastries, Buns, Cakes and gateaux, Biscuits—chocolate, Other biscuits—digestive and ginger.
Non-starchy vegetables	Vegetables, Tinned vegetables, Carrots, Parsnips, swede and turnip, Sweet-corn and mixed veg, Tomatoes, Spinach, Broccoli, Brussels sprouts and spring greens, Cabbage and cauliflower, Peppers and watercress, Onion, Green salad, Side salads in dressing, Courgettes, marrow and leeks, Mushrooms, Vegetable dishes.
Legumes	Peas and green beans, Beans and pulse.
Fruits	Fruit puddings, Tinned fruit not including grapefruit, prunes, figs or blackcurrants, Cooked fruit not including blackcurrants, Dried fruit, Fresh apples and pears, Fresh oranges and orange juice, Grapefruit and grapefruit juice, Blackcurrants, Ribena and hi-juice blackcurrant drinks, Other fruit juices (not squashes), Bananas, Fresh peaches, plums, cherries and grapes, Strawberries and raspberries, Fresh pineapple, melon, kiwi fruit and other tropical fruit.
Nuts	Hazelnuts, Cashews, Almonds, Brazil nuts, Walnuts.
Meat	Bacon and gammon, Pork, Lamb, Beef, Minced meat dishes, Meat pies, Liver and kidney, Pate and liver sausage, Faggots and black pudding, Sausages, Ham and luncheon meat, Chicken and turkey.
Fish	White fish, Fish fingers and fish dishes, Oily-fish, Shellfish.
Dairy Products	Cottage Cheese, Cheese, Pizza, quiches and cheese flans, Milk based puddings and sauces, Yogurt and fruit fools, Ice cream and chocolate desserts, Cream, Drinking chocolate and milk shakes not including McDonald style milkshakes.
MUFA+PUFA: SFA	Ratio of monounsaturated lipids and polyunsaturated lipids to saturated lipids.
Eggs	Mayonnaise/salad cream, Boiled/poached eggs, Omelette and fried eggs.
Potatoes	Potatoes boiled/jacket, Roast potatoes/chips, Crisps savoury snacks.
Sweets	Added sugar, chocolate, other sweets and sweet spreads.
Drinks	Diet coke/Pepsi not caffeine free, Coke and Pepsi, Soft drinks not diet drinks, drinking chocolate/milk shakes, decaffeinated coffee/tea, tea and coffee.
Miscellaneous	Gravy granules and powders, pickles chutney, ketchup, brown sauce, stock cubes, marmite, soup.
